# Molecular Cloning and Analysis of the *Tryptophan oxygenase* Gene in the Silkworm, *Bombyx mori*

**DOI:** 10.1673/031.008.5401

**Published:** 2008-09-23

**Authors:** Liu Yan, Meng Zhi-Qi, Niu Bao-Long, He Li-Hua, Weng Hong-Biao, Shen Wei-Feng

**Affiliations:** ^1^ Sericulture Research institute, Zhejiang Academy of Agricultural Sciences, Hangzhou, 310021, China

**Keywords:** eye pigmentation, Lepidoptera, *vermilion gene*

## Abstract

A *Bombyx mori* L. (Lepidoptera: Bombycidae) gene encoding tryptophan oxygenase has been molecularly cloned and analyzed. The tryptophan oxygenase cDNA had 1374 nucleotides that encoded a 401 amino acid protein with an estimated molecular mass of 46.47 kDa and a PI of 5.88. RT-PCR analysis showed that the *B. mori tryptophan oxygenase* gene was transcribed in all examined stages. Tryptophan oxygenase proteins are relatively well conserved among different orders of arthropods.

## Introduction

The silkworm. *Bombyx mori* L. (Lepidoptera: Bombycidae), is an important agriculturally insect that has been domesticated and used in silk production for about 5000 years. As a key model insect for the Lepidoptera, which include many destructive agricultural pests (Tomita et al. 2003), analysis of the genome of the silkworm is important.

The pigments present in the insect eye, usually both pteridine and ommochrome, determine its color (Summers et al. 1982). In *Drosophila*, a large number of mutations are known to affect the pigmentation of the compound eye, including those that affect the biosynthesis or transport of ommochrome (brown) and pteridine (red) pigments (Beadle and Ephrussi 1937). Ommochrome has been recently demonstrated to contribute to eye coloration in mosquitoes and beetles (Beard et al. 1995; Lorenzen et al. 2002; Fabrick et al. 2004). One of the genes involved in the ommochrome biosynthetic pathway is tryptophan oxygenase (TO), an enzyme that converts tryptophan to N-formylkynurenine which is then converted to kynurenine by kynurenine foramidase (Ferre et al. 1985; Takikawa et al. 1986). Tryptophan catalyzes the first step in the synthesis of the brown eye pigment of the fly (Linzen et al. 1974; Walker et al. 1986) and is an essential amino acid that is required in several physiological processes in addition to protein synthesis. Early work on *Drosophila* eye color mutants revealed that *vermilion* (*v*) and *cinnabar* (*cn*) are involved in ommochrome production (Beadle and Ephrussi 1937). The *vermilion* gene has been shown to encode tryptophan oxygenase and has been found to be a potentially useful germline transformation marker (Lorenzen et al. 2002). The *tryptophan oxygenase* gene has also been identified from *Anopheles gambiae* (Mukabayire et al. 1996), *Aedes aegypti* (Fang and Li 2001), *Tribolium castaneum* (Lorenzen et al. 2002), *Plodia interpunctella* (Fabrick et al. 2004), and *Shistocerca americana* (Dong and Friedrich 2005).

Abraham et al. (2000) examined the *ABC transporter* genes in *B. mori* that are among the eye- and egg-color mutations affecting the synthesis and accumulation of ommochrome pigments in *B. mori*. They are homologous to the *Drosophila white* gene, and are involved in transporting pigment precursor (Abraham et al. 2000). The protein sequence of *B. mori* kynurenine 3-monooxygenase showed high identity with *cinnabar* (*cn*) in *Drosophila* (Quan et al. 2001; Lorenzen et al. 2002). Yet little is known about the *vermilion* homologous gene in *B. mori*. Here, the *B. mori tryptophan oxygenase* gene was cloned and analyzed.

## Materials and Methods

### Insects feeding and sample collection

Larvae of the normal strain (*Daizo*) were reared routinely on mulberry leaves. The temperature ranged from 25 to 27 °C and humidity varied between 70 and 80%.

### Preparation of RNA and RT-PCR

Total RNA was separately extracted from samples using Trizol. RNA was reverse-transcribed by using oligo (dT)18 adaptor primer (Sangon Bio, www.sangon.com) and avian myeloblastosis virus reverse transcriptase (Promega, www.promega.com) at 42 °C for 60 min. cDNA fragments were amplified using Ex Taq (Takara Bio, www.takara-bio.com) with the following degenerate primers 5′ TAY GAR YTN TGG TTY AAR CA 3′ (Fto 1, sense) and 5′ CAT TKC KTT GCA CCA TSA WMA CRT GAT T 3′ (Rtol, antisense). Thermal cycling conditions were as follows: 94 °C for 5 min; 30 cycles at 95 °C for 30 s, 50 °C for 30 s, and 72 °C for 1min. The last cycle was followed by an extension at 72 °C for 10 min. Amplified products were separated by gel electrophoresis on 1.2% agarose gels at 100 v for approximately 1 h using a 1 × TAE buffer (40 mM Tris acetate and 2 mM EDTA in water). After electrophoresis, the gel was stained for 30 min in 0.01% SYBRTM Green I nucleic acid gel stain (FMC Bio, www.fmc.com). Following purification of PCR products by Biospin Gel Extraction Kit (BioFlux Bio, www.fluxionbio.com), the purified PCR fragments were TA-cloned into pMD18-T (Takara Bio.). The insertions were identified by PCR amplification using M13 forward and reverse primers before sequencing. No PCR products were produced when cDNA template was excluded during reverse transcription polymerase chain reaction.

### RACE method

Rapid amplification of cDNA ends (RACE) method was applied to obtain full-length cDNAs. cDNAwas synthesized from 2 µg of total RNA using BD SMART RACE cDNA Amplification Kit (BD Biosciences, www.bdbiosciences.com). The 5′ and 3′ cDNA ends were obtained by touchdown PCR with LA Taq (Takara Bio) using a universal primer mixture (UPM) and the following gene-specific primers (GSPs): 5′ GTA ATG CCC TCA CAG AAT CCA CTT 3′ (Bmtoa, 5′RACE) and 5′ GTC TCA TCA CGA AAT GGC GTT ACA 3′ (Bmtob, 3′RACE) according to manufacture's instructions. RT-PCR products were gel purified, cloned into pMD18-T (Takara Bio) and sequenced.

To acquire the integral cDNA of *Bmto*, primer 5′ CAT TGA AAT GGC GTG TCC TAT GAG 3′ (Fto2, Sense) and 5′ GTT ATA AAG AAG CTT CAA GGC CGT 3′ (Rto2, Antisense) for the gene encoding the *B. mori* homolog of the tryptophan oxygenase were used in amplification. The PCR product was subcloned into pMD18-T and sequenced.

### Analysis of the expression at different developmental stages

For developmental analysis, total RNA was extracted from various stages of development (egg, third instars, fifth instars, pupae and adult). RT-PCR was performed as described above. Real-time PCR was performed using the SYBR premix EX Taqtm kit (Takara Bio). The primers designed for *Bmto* were 5′ ACA CGC ACG GGT TCA ACT TCT 3′ (FBmto-real, Forward) and 5′ ATG TGA CAG CCT CCT TTC TCC T 3′ (RBmtoreal, Reverse), and for the *B. mori* Actin 1 that was used as internal control were 5′ ACC CAT CTA CGA AGG TTA CGC 3′ (FBmActin, Forward) and 5′ ACG AAC GAT TTC CCT CTC AGC 3′ (RBmActin, Reverse), yielded 212 and 142 bp bands, respectively. PCR amplification and fluorescence detection were performed using the DNA Engine Option 2 under the following thermal cycle conditions: 95 °C for 1 min, 45 cycles of 95 °C for 10 s, 60 °C for 20 s. To reach reproducibility, each sample was performed three times. Ct values of *Bmto* were calculated to the actual concentrations based on the standard curve. *BmActin* transcripts were used to standardize the different *Bmto* cDNA samples.

### Sequence analysis

Predictions of isoelectric point and molecular weight were carried out at http://cn.expasy.org. The amino acid sequence of BmTO was submitted to predict secondary structure at http://npsa-pbil.ibcp.fr and conserved protein domain at http://www.ncbi.nlm.nih.gov/. Alignment of deduced amino acids from cDNA clones was made using DNAMAN software. A phylogenetic tree based on deduced amino acid difference was constructed by NJ (Neighbor-joining) method using PHYLIP (http://bioweb.pasteur.fr/seqanal/interfaces/protpars.html). Reliability of the NJ tree was assessed by interior branch test, using 1000 replications.

## Results

### Identification of cDNA sequences encoding BmTO

**Figure 1.**
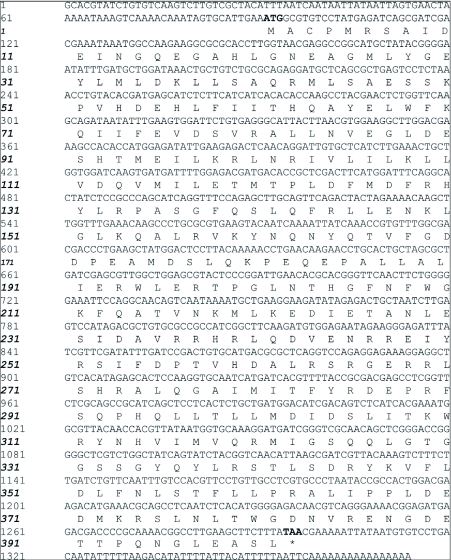
The nucleotide and deduced amino acid sequence of *Bmto* cDNA. Nucleotides are numbered on the left of each line. The deduced amino acid sequence is shown below the nucleotide sequence and numbered from the first methionine. The primer sites are indicated by arrows. The amino acid of conserved tryptophan oxygenase domain is shadowed. Initiation and termination codons are shown in bold face.

Using the degenerate primers, a partial *Bmto* cDNA was obtained. RACE was used to complete the missing 5′ and 3′ ends of the cDNA. The cDNA consisted of 1374 bp long with an open reading frame of 401 amino acids (Figure 1). BmTO (*B. mori* tryptophan oxygenase) was assigned its name because of its similarity to the known tryptophan oxygenase protein.

### Bmto expression profile in B.mori larvae

**Figure 2.**
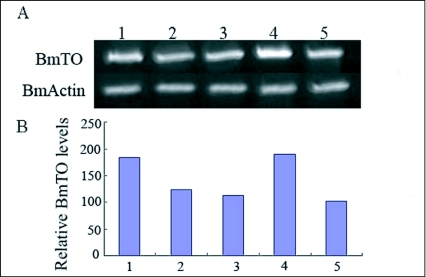
Expression of *Bmto* gene in different stages. RT-PCR results of five stage samples are shown in A, which is amplified after 25 cycles. Real time RT-PCRs were performed with specific primer pairs for the Bmto or *actin-l* and shown in B. Vertical scales show the relative molecular weight of *Bmto* relative to *Actin-l*. Horizontal data 1–5 represented the five different stages of embryos, third instar larvae, fifth instar larvae, pupae and moth, respectively.

To examine the expression of *Bmto* gene in various life stages, real time RT-PCR using total RNA obtained from different instars was performed. *Bm actin* was used as an internal control. The level of *Bmto's* expression was quantified by calculating the ratio of *Bmto*/*BmActin* of the same sample. As shown in Figure 2, *Bmto* was expressed in all samples examined. Transcript levels were low in larvae and adults, and much greater in embryos and pupae.

### Comparison of BmTO to other tryptophan oxygenase proteins

**Figure 3.**
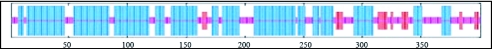
Secondary structure of the *Bombyx* *mori* tryptophan oxygenase determined by http://npsa-pbil.ibcp.fr. Alpha helices are shown in blue, β-sheets in red and random coils in magenta.

The predicted molecular weight and pi of *B. mori* tryptophan oxygenase were 46.47 kDa and 5.88, respectively. BmTO was not predicted to be a secreted protein as determined by SignalP. There was 60.85% alpha helix, 30.92% random coil and 8.23% extended strand in the secondary structure of BmTO (Figure 3).

**Figure 4.**
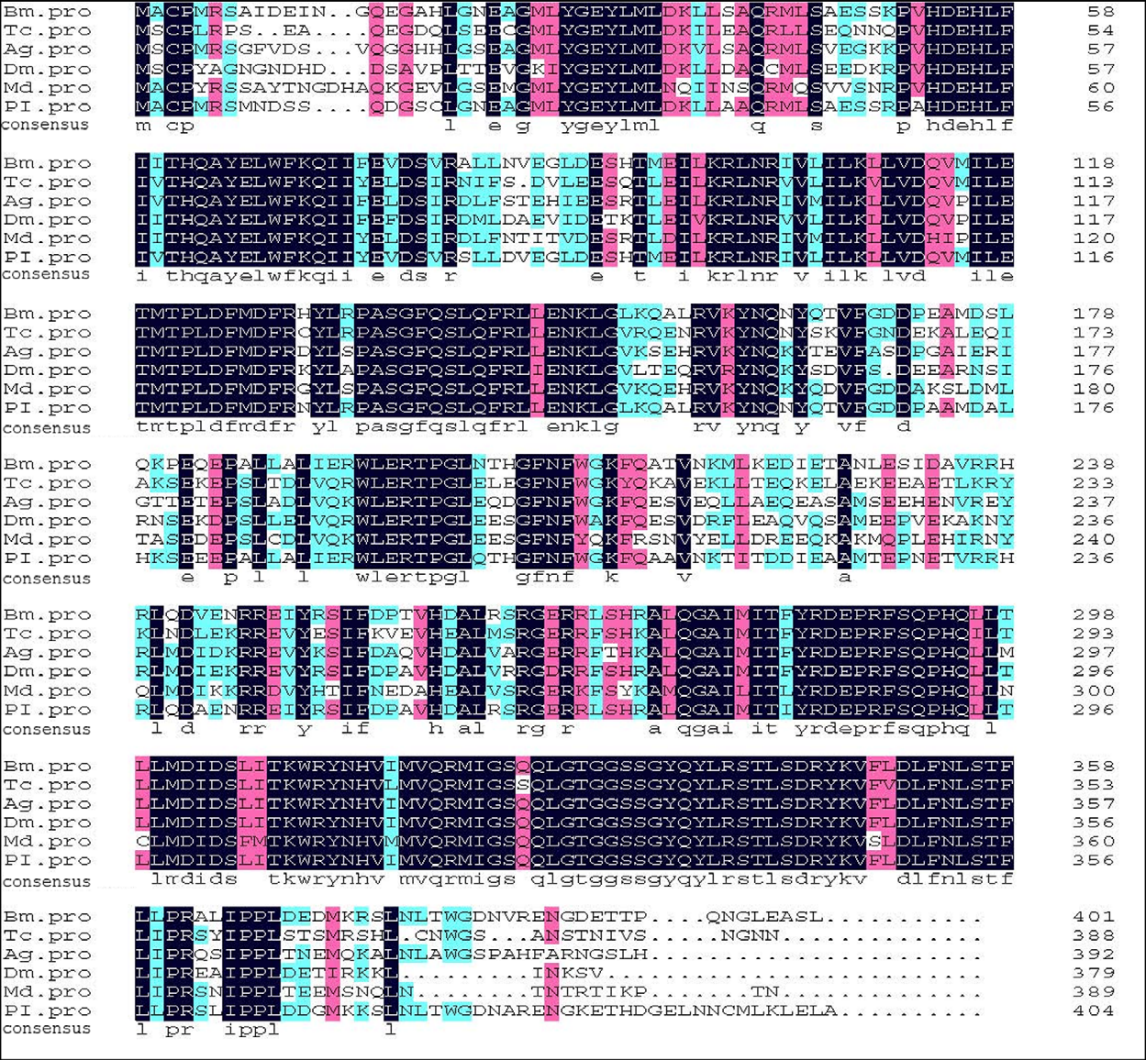
Sequence alignment of tryptophan oxygenase proteins from *Bombyx mori, Tribolium castaneum* (Tc, NP_001034499), *Anopheles gambiae* (Ag, XP_312204), *Drosophila melanogaster* (Dm, NP_511113), *Mayetiola destructor* (Md, ABC69733) and *Plodia interpunctella* (Pi, AAR24625). Completely conserved residues are indicated below the alignment.

**Figure 5.**
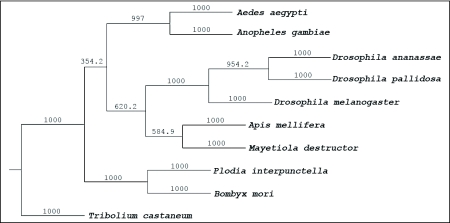
Phylogenetic tree of *Bombyx mori* tryptophan oxygenase with other family members. The distance tree was calculated using the MEGA program, which was based on a Clustal alignment of the sequences after phylogenetic analysis. Branch lengths are proportional to percentage sequence difference.

To assess the relatedness of the BmTO to tryptophan oxygenase proteins from other species, identities were calculated based on a Clustal alignment including nine tryptophan oxygenase protein sequences (Figure 4). Amino acid comparisons revealed 87.2% identity between the deduced protein of BmTO and previously known tryptophan oxygenases from the moth, *Plodia interpunctella* (Figure 4, 5). Identity of tryptophan oxygenase proteins was high within the arthropods (at least 65.1%), which verified that *tryptophan oxygenase* gene was relatively conserved among the insects.

## Discussion

Eye pigments of *D. melanogaster* were determined by specific enzymes of biosynthesis, ATP binding cassette transporters of precursors of pigments and pigment granules (Lloyd et al. 1998). Tryptophan oxygenase is an essential amino acid of insects as its absence or deficiency results in eye color defects in *Drosophila* and other insect species (Summers et al. 1982; Miyashita et al. 1994; White et al. 1996; Lorenzen et al. 2002; Fabrick et al. 2004). In the present study, the *Bombyx mori* tryptophan oxygenases homolog (*Bmto*) was successfully cloned and analyzed. *Bmto* was expressed at a wide variety of developmental stages, which was consistent with that of ommochromes (Sawada et al. 2000). BmTO is highly similar to other tryptophane oxigenases, including those regions of the protein that are constrained by functional requirements among diverse species.

Dominant phenotypic markers such as eye color genes can be used in the development of transgeneic organisms. These genes are not limited by cell autonomy and generate an easily scored visible phenotype when introduced into the appropriate mutant background. The use of such genes eliminates the need for specialized detection systems, thus making transformation-based protocols more widely accessible. Fridell and Searles (1991) constructed a germ line transformation vector that utilizes the *vermilion* as the selectable maker gene, which encodes the enzyme tryptophan oxygenase in *Drosophila*. Loukeris et al. (1995) developed a transformation system for the medfly *Ceratitis capitata* with the eye color gene white. White et al. (1996) linked *vermilion* cDNA of *D. melanogaster* to the inducible hsp82 promoter of *D. pseudoobsura*. This marker rescued adult eye color in a strain of *Musca domestica* that was homozygous for a mutant tryptophane oxygenase gene (White et al. 1996). Besansky et al. (1997) placed *Anopheles gambiae* cDNA encoding tryptophan oxygenase under the control of the constitutive baculovirus promoter, *ie-1*. This chimeric construct, expressed transiently in *vermilion* mutants of *D. melanogaster*, partially rescued adult eye color (Besansky et al. 1997). Genes such as *Bmto* may prove invaluable as transformation markers in the future.

## Abbreviations

**BMTO** -: Bombyx mori tryptophan oxygenase
